# Fabrication of an Electrically-Resistive, Varistor-Polymer Composite

**DOI:** 10.3390/ijms131215640

**Published:** 2012-11-23

**Authors:** Mansor Bin Ahmad, Asma Fatehi, Azmi Zakaria, Shahrom Mahmud, Sanaz A. Mohammadi

**Affiliations:** 1 Department of Chemistry, Faculty of Science, Universiti Putra Malaysia, 43400 Serdang, Selangor, Malaysia; E-Mail: s.abdolmohammadi@yahoo.com; 2 Department of Physics, Faculty of Science, Universiti Putra Malaysia, 43400 UPM Serdang, Selangor, Malaysia; E-Mail: azmizak@gmail.com; 3 Nano Optoelectronic Research & Technology (NOR) Lab, School of Physics, Universiti Sains Malaysia, 11800 Minden, Pulau Pinang, Malaysia; E-Mail: shahromx@usm.my

**Keywords:** polymer composite, nano-metric varistor powder, polycaprolactone, electrical resistance

## Abstract

This study focuses on the fabrication and electrical characterization of a polymer composite based on nano-sized varistor powder. The polymer composite was fabricated by the melt-blending method. The developed nano-composite was characterized by X-ray diffraction (XRD), transmission electron microscopy (TEM), field emission scanning electron microscopy (FeSEM), and energy-dispersive X-ray spectroscopy (EDAX). The XRD pattern revealed the crystallinity of the composite. The XRD study also showed the presence of secondary phases due to the substitution of zinc by other cations, such as bismuth and manganese. The TEM picture of the sample revealed the distribution of the spherical, nano-sized, filler particles throughout the matrix, which were in the 10–50 nm range with an average of approximately 11 nm. The presence of a bismuth-rich phase and a ZnO matrix phase in the ZnO-based varistor powder was confirmed by FeSEM images and EDX spectra. From the current-voltage curves, the non-linear coefficient of the varistor polymer composite with 70 wt% of nano filler was 3.57, and its electrical resistivity after the onset point was 861 KΩ. The non-linear coefficient was 1.11 in the sample with 100 wt% polymer content. Thus, it was concluded that the composites established a better electrical non-linearity at higher filler amounts due to the nano-metric structure and closer particle linkages.

## 1. Introduction

Polymer-matrix composites with discontinuous fillers (particles) are widely used in electronics [1]. The terms “filler” and “functional filler” refer mostly to short, discontinuous fibers, flakes, platelets, or particles. Inorganic reinforcing fillers are stiffer than the matrix and deform less, causing an overall reduction in the matrix strain, especially in the vicinity of the particle as a result of the particle/matrix interface [2]. In the case of a conducting composite, the greater the volume fraction of the conducting filler, the higher the conductivity of the composite will be, since the polymer matrix is usually insulating. The matrix used in making a polymer-matrix composite can be in liquid or solid form during the mixing of the matrix and the filler. In the resulting composite, the percolation attained after mixing the matrix and the filler through subsequent composite fabrication means they are involved in the flow of the thermoplastic under heat and pressure. In percolation, the filler units touch one another to form continuous paths; however, there is considerable contact resistance at the interface between the touching filler units [3]. An effective way to decrease this contact resistance is to bond the filler units together at their junctions by using a solid that melts and wets the surface of the filler during the fabrication of the composite. The low-melting-point solid can be in the form of particles that are added to the composite mix, or it can be in the form of a coating on the filler units.

It is important to note that the use of a non-linear, conducting filler will result in interesting and useful effects on the polymer composite [4–9]. This functional filler, which is made of doped zinc oxide (ZnO), disperses throughout the polymer matrix and responds to the electrical, non-linear behavior at room temperature [10–13]. Due to the small sizes of the nano particles relative to the micron-sized fillers, the nano particles have a much higher interfacial area per unit volume. The reduction in particle size improves the overall properties of a varistor. Nanoparticles yield a narrow grain-size distribution and withstand relatively high energies [14]. In order to take advantage of these effects, the nano fillers must be well dispersed in the polymer matrix.

Extensive research has been done on the multi-functional, inorganic nanoparticle, nano ZnO, due to its many significant physical and chemical properties. Tang and co-workers studied the UV-shielding properties of nano ZnO/Polymethyl methacrylate composites in 2005 [15]. They found that increasing the amount of nano ZnO improved the UV-shielding capability of the polymer composite. Ultraviolet absorption, thermal behavior, and the visco-elastic properties of nano ZnO in Polyvinyl alcohol/Polyethylene oxide was investigated by Lee *et al.* in 2008 [16]. Photo-degradation of low density polyethylene (LDPE) containing ZnO nano particles was studied by Yang *et al.* in 2010 [17]. Raju *et al.* investigated the improved and best tribological behaviors of polyester filled with ZnO nano particles [18]. It was demonstrated that the improved tribo-performance of the nano composite was attributable to the mechanical properties of the nano particles. In addition, ceramic fillers, such as ZnO, have well-known, non-linear behaviors, which were studied by Wang *et al.*[19]. ZnO/EPDM (ethylene propylene diene monomer) nanocomposites were processed by melt blending in their research. As a result, the geometry, as well as the intrinsic, non-linear behavior of the filler, impacted the non linearity of the composites.

ZnO varistor was announced in 1969 by Matsuoka [20], although some such development was conducted in Russia in the early 1950s [21]. A more-detailed paper provided by Matsuoka in 1971 described many of the essential features of varistors as we know them today. The details included ZnO semiconductors with the addition of substituted ions, densification by liquid-phase sintering with a Bi_2_O_3_-rich liquid phase, and segregation of large ions to the grain boundaries. ZnO-based varistors, as one type of metal-oxide varistors, are produced by a ceramic sintering process that produces a structure of conductive grains that are composed of the matrix oxide surrounded by electrically-insulating barriers. These electrical barriers are derived from trap states at the grain boundaries that are induced by the additive elements [22–25]. The milling and homogenization stages of the powders are conducted mainly in a ball mill in an aqueous mixture. Pure oxides are used as dopants in the zinc oxide ceramic varistor, including Bi_2_O_3_, Co_3_O_4_, MnO, and others. The density of traditional ZnO-based polycrystalline ceramics is generally increased by the presence of Bi_2_O_3_, which forms a liquid phase during the sintering stages. Other dopants, such as Co_3_O_4_ and MnO, are added in order to increase the value of the nonlinear coefficient (alpha) and the resistance against degradation [26]. High requirements are imposed on the initial materials, both with respect to purity and dispersion composition, so as to obtain a homogenous microstructure of the sintered body. It was found that the dopants that were added to ZnO affected the formation of the microstructure and, consequently, affected the electrical and other performance properties of the varistors differently [27,28]. Dopants are divided into three basic groups according to the functional applications, as follows:

- Those that participate in the formation of the basic microstructure of ZnO varistors in sintering provide for the formation of inter-granular layers; Bi_2_O_3_ is one such dopant.
- Those used in ensuring the non-linearity of the varistor ceramic promote the creation of deep charge carrier traps and cause the formation of the surface potential of the grains; Co_3_O_4_ and MnO are such dopants.
- Those that stabilize inter-granular layers under electrical loads and external environmental factors (temperature and humidity) and increase the stability of the electrical characteristics and reliability of the varistors; Sb_2_O_3_ is one such dopant [29].

Polycaprolactone (PCL) is a hydrophobic, semicrystalline polymer whose crystallinity tends to decrease with increasing molecular weight. The good solubility of PCL and its exceptional blend-compatibility have stimulated extensive research into the potential application in the biomedical field [30–35]. Its numerous advantages over other polymers include tailorable degradation kinetics and mechanical properties, ease of shaping and manufacture enabling appropriate pore sizes conductive to tissue in-growth, and the controlled delivery of drugs contained within their matrix. Moreover, functional groups can also be added to render the polymer to become more hydrophilic, adhesive or biocompatible which enables favorable cell responses due to the fact that PCL degrades at a slower rate (*i.e.*, up to 3–4 years).

Although it initially attracted some research interest, PCL was soon overwhelmed by the popularity of other resorbable polymers, such as polylactides and polyglycolides. Furthermore, both the medical-device and drug-delivery communities considered that faster resorbable polymers also have fewer perceived disadvantages associated with long-term degradation and intracellular resorption pathways. This consequently caused PCL to become almost forgotten for most of the last two decades. In the 1970s, it was already recognized that PCL was particularly amenable to blending and polymer blends based on PCL were categorized with three types of compatibility; these were exhibiting only a single *T*_g_, as mechanically compatible while exhibiting the *T*_g_ values of each component, but with superior mechanical properties and as incompatible, *i.e.*, exhibiting the enhanced properties of phase-separated materials [35]. The compatibility of PCL with other polymers depends on the ratios employed, and PCL is generally used to produce better control over the permeability of delivery systems. Copolymers of PCL can be formed using many monomers, such as ethylene oxide, polyvinylchloride, chloroprene, polyethylene glycol, polystyrene, diisocyanates, tetrohydrofuran, diglycolide, dilactide, substituted caprolactone, methyl methacrylate and vinyl acetate [30], which dictate the crystalline nature of PCL that enable its easy formability at relatively low temperatures.

Meanwhile, the physico-mechanical properties of several degradable polymers of PCL were studied and compared by Engelberg and Kohn who investigated thermal and tensile properties, including Young’s modulus, tensile strength, and elongation at yield and break [36].

PCL is prepared by the ring-opening polymerization of the cyclic monomer, e-caprolactone, and it has been studied since the 1930s [37]. Various catalysts, such as stannous octoate, have been used to catalyze the polymerization, whereas low-molecular-weight alcohols can be used to control the molecular weight of the polymer [38]. There are various mechanisms that affect the polymerization of PCL, *i.e.*, anionic, cationic, co-ordination, and radical mechanisms. In particular, each method affects the resulting molecular weight, molecular weight distribution, end-group composition, and the chemical structure of the copolymers [34]. The average molecular weights of PCL samples generally vary from 3000 to 80,000 g/mol and can be graded according to their molecular weights [39]. Based on the degradation studies presented in the literature, it can be concluded that PCL undergoes a two-stage degradation process, namely, the non-enzymatic hydrolytic cleavage of ester groups and, when the polymer is more highly crystalline and has a low molecular weight (*i.e.*, less than 3000), the polymer has been shown to undergo intracellular degradation as evidenced by the observation of the uptake of PCL fragments by the phagosomes of macrophages and giant cells and within fibroblasts [40]. This supports the theory that PCL may be resorbed completely and degraded via an intercellular mechanism once the molecular weight is reduced to 3000 or less. It is also important to note that, in the first stage, the degradation rate of PCL is essentially identical to the *in vitro* hydrolysis at 40 °C and obeys first-order kinetics. Thus, it was concluded that the mechanism of PCL degradation could be attributed to the random hydrolysis chain scission of ester linkages, causing a decrease in the molecular weight. Flexibility, biodegradability, low *T*_g_ (−61 °C) and a fairly long biodegradability cycle have been noted as particular features of PCL [41], so that, when it is mixed with an inorganic filler, the overall properties of the composite are enhanced.

This study introduced an electrically-resistive, polymer composite with varistor-like behavior, devoted to the increased interest in the varistor-based composite. Here, we determined the morphology and the current-voltage characteristics of composites that are made up of various weight percentages of varistor powder as nano filler in the PCL.

## 2. Results and Discussion

### 2.1. X-ray Diffraction

**Figure 1:**
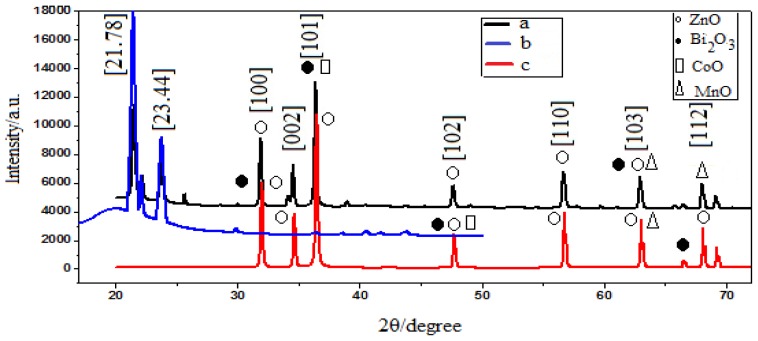
X-ray diffraction (XRD) patterns of (a) varistor polymer nanocomposite composite; (b) Polycaprolactone (PCL) polymer; (c) nano-sized varistor powder.

Figure 1 shows the typical X-ray diffraction (XRD) patterns of PCL polymer, varistor powder, and the varistor polymer composite that contained 70 wt% of varistor powder. PCL is a semi-crystalline material that shows a strong diffraction at a 2θ of 23.44° and a weak diffraction at 21.78°, indicating highly-ordered, chain-folding characteristics at a maximum intensity of 17,000, as shown in Figure 1a[42]. The composite had seven major peaks at peaks of 2θ of varistor components at 31.87°, 34.57°, 36.30°, 47.63°, 56.67°, 62.94°, and 67.98°, which were indexed as 100, 002, 101, 102, 110, 103, and 112, respectively. The varistor polymer composite had diffraction peaks that were approximately similar to the peaks of the varistor components without PCL peaks (Figure 1a,c). In the composite, the intensity reduction of PCL may be assigned to the increased peak intensity of the fillers due to its high percentage [42]. It was noted that the peaks corresponding to 100, 002, 101, 102, 110, 103 and 112 planes in the mixture of PCL with nano-filler powder spectrum, come from the ZnO crystals. The crystallographic planes of 101, 102, and 103 were assigned to Co_3_O_4_ (hexagonal), Bi_2_O_3_ (monoclinic) and MnO (orthorhombic), respectively. Two patterns of the nanocomposite and varistor powder showed strong diffractions of the ZnO crystal. This indicated that the four main components (ZnO, MnO, Bi_2_O_3_ and Co_3_O_4_) formed secondary phases, namely, ZnMn_2_O_4_ (ICSD code: 077047), ZnCo_2_O_4_ (ICSD code: 011149), and Zn_0·33_Bi_12·67_O_19·33_ (ICSD code: 800822), that produce the non-linear I–V behavior of the varistor composite, due to the substitution of Zn with other cations, such as Bi, Mn, and Co. Table 1 shows a comparison of the peak positions of secondary phases that were formed, both in the nano-sized varistor powder and the varistor polymer composite. The ZnO particles were surrounded by a thin layer that was enriched with the cations of the additives [43,44]. When the diffraction pattern of the filler was compared to that of the mixture of the PCL/filler powder, it was evident that the crystallinity had changed.

The changes in the XRD patterns clearly indicated coordination between the components of the composite [45]. According to the XRD patterns, it was noted that the diffraction peaks in the case of the PCL/nano varistor composite had moved to a slightly lower value of 2θ, since the concentration of the nano varistor powder was high in the composite.

### 2.2. Electron Microscopic Analysis

**Figure 2:**
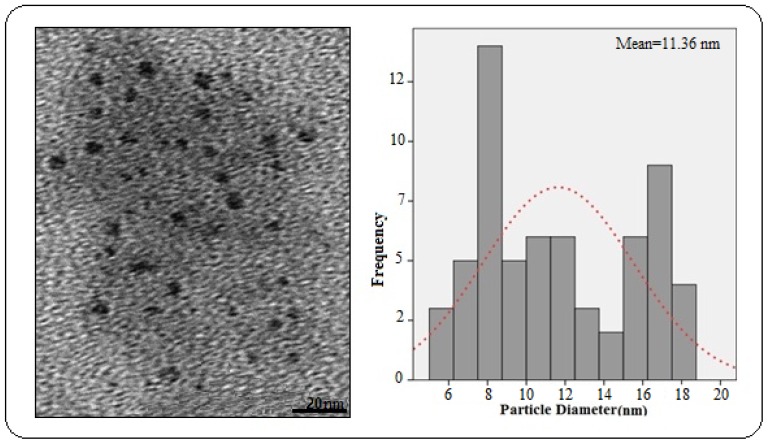
The transmission electron microscopy (TEM) image and the particle size distribution for polymer nanocomposite with 70 wt% nano varistor powder.

Figure 2 presents the transmission electron microscopy (TEM) image and the particle-size distribution of the nano-sized varistor polymer composite. The TEM image showed that the nano powder filler particles throughout the matrix were almost spherical, and, from the histogram of the normal particle-size distribution, the mean particle diameter was determined to be 11.36 nm. The TEM samples were prepared by dispersing the polymer nano-composites in chloroform, and the solution was drop-cast onto carbon-coated copper.

**Figure 3:**
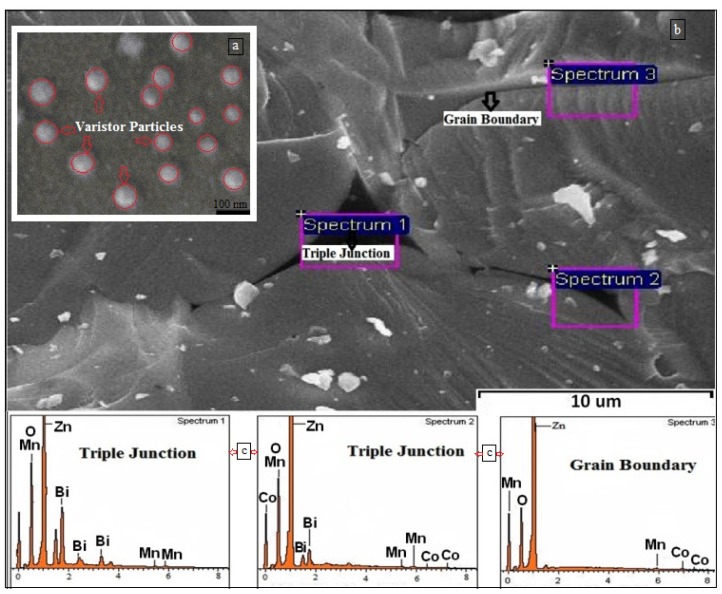
Field emission scanning electron micrograph (FeSEM) image of varistor powder at 1280 °C, 2.5 h (**a**,**b**) and energy dispersive X-ray (EDAX) micrograph of varistor powder at grain boundaries and triple junction (**c**).

It is important to note that the non-linear, I–V behavior of the varistor composite originated from a grain boundary effect. From the field emission scanning electron micrograph (FeSEM) (Figure 3a,b), it can be seen that the ZnO-doped particles had contacts and, because of the high sintering temperature, holes were also observed. In order to help better investigate the structure of the grain boundary at the contact points of the particles, a FeSEM micrograph was taken of the non-grinding powder (Figure 3b). The importance of the presence of dopants (Bi, Mn, Co) on the grain boundaries to gain current-voltage characteristics was emphasized by energy dispersive X-ray (EDAX) spectroscopy (Figure 3c). This indicated that the grain boundary was composed of ZnO and small amounts of Mn and Co in triple junctions, as well as grain boundary and a small amount of Bi in the triple junctions. The presence of Mn and Co indicates that the Mn and Co ions were substituted in the Zn lattice. It is for this reason that the ionic radii of Mn and Co ions are smaller than the Zn radii in the grain boundaries [46].

### 2.3. Electrical Measurement

**Figure 4:**
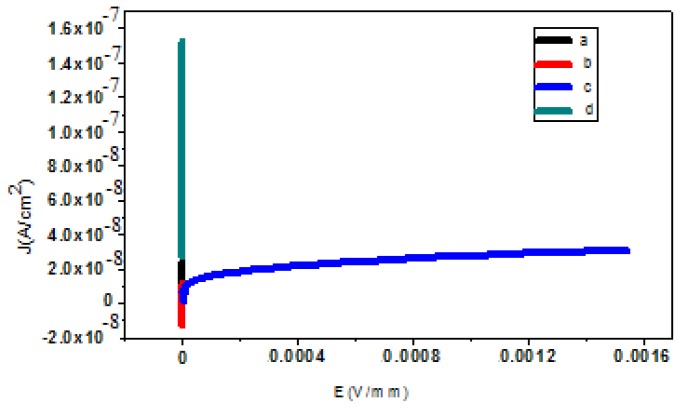
(a) The non-linear characteristic of polymer composites with 15 wt%; (b) 20 wt%; (c) 70 wt% nano-sized varistor powder; (d) neat polymer (100 wt% PCL).

The I–V characteristics for nano varistor polymer composites are shown in Figure 4. The I–V behavior is linear at small voltages, but it becomes non-linear above a certain voltage, which is the switching voltage of the device. From these curves, the following conclusions can be drawn.

For the nano varistor polymer composite, as the concentration of filler increases from 15 wt% (Figure 4a) to 70 wt% (Figure 4d), the alpha value increases from 1.71 to 3.57. The increase of the non-linear coefficient was attributed to the increase of the embedded amount of nano filler. The non-linear coefficients for four different concentrations of polymer composites are presented in Table 2. As shown in the table, the varistor polymer composite with 20 wt% of nano filler (Figure 4b) exhibited very poor properties. It was assumed that the highly non-linear properties of the varistor polymer composite with 70 wt% of nano filler were attributable to excess nano-particle linkages. The onset voltage and onset current were also determined. As an example, the onset voltage and onset current for the varistor polymer nanocomposite with 70 wt% of nano filler were 11.96 V and 3.52 × 10^−5^ μA. It is well-known that varistor is a kind of resistor with non-ohmic current-voltage characteristics. The resistance parameter for the varistor polymer nanocomposite with a high percentage of nano filler was approximately 49 KΩ at the onset point. The resistivities before and after the onset point were 758 KΩ and 861 KΩ, respectively.

The non-linear coefficient of the ceramic varistor from the I–V characteristic can be calculated from the reciprocal slope of the I–V curve in the non-linear region using the following expression:

$$\alpha =\frac{\text{log }I_{2}-\text{log }I_{1}}{\text{log }V_{2}-\text{log }V_{1}}$$

where *I*_1_ = 1 mA, *I*_2_ = 10 mA, *V*_1_ and *V*_2_ are the voltages corresponding to *I*_1_ and *I*_2_, respectively.

## 3. Experimental Section

### 3.1. Materials

Commercial-grade poly(ɛ-caprolactone), PCL, was purchased from Solvay (Warrington, England), while the dopant oxides, Bi_2_O_3_ (99.9 wt% pure), Co_3_O_4_ (99.9 wt% pure), and MnO (99.9 wt% pure) were purchased from Alpha Aesar (Karsluhe, Germany); the nano ZnO powder (>99 wt% pure) was purchased from ICI (Tehran, Iran).

### 3.2. Preparation ZnO Based Nano-Sized Varistor Powder

The first steps in producing fine varistor powder are similar to the process developed by Wang *et al.*[47]. The ZnO-based varistor powder that was prepared in the present study contained 98.5 mol% of nano powder ZnO, 0.5 mol% of Bi_2_O_3_, 0.5 mol% of MnO, and 0.5 mol% of Co_3_O_4_. A mixed water solution containing the required amounts of metal oxides (except ZnO) was ball-milled with zirconium balls to obtain a fine powder. After milling for about 1 h, nano ZnO powder was added to the mixture and it was kept in the ball mill with a constant milling rate for another hour. The slurry was dried at 150 °C for 8 h, ground into powder, and calcined at 750 °C in air. After calcination, the filler was ground and then sintered at 1280 °C for 2.5 h. Mechanical activation by grinding can enhance chemical homogeneity and is a critical factor in determining electrical properties [48]. For this reason, the varistor powder used in this study was ground before it was embedded in the polymer matrix.

### 3.3. Fabrication of Varistor Polymer Nanocomposite

A polymer composite was fabricated by the melt-blending method using a Thermo HAKKE chamber. PCL granules were placed in the chamber before it was heated and stabilized at 70 °C. Next, the filler was gradually added to the melted PCL within 15–60 min to gain a polymer composite with well-dispersed filler. The polymer composite was held in contact with the heated plates of the hot press at a pressure of 110 kg/cm^2^ for 5 min and then exposed to a cold press at room temperature for 10 min at the same pressure to form 80 cm × 80 cm plaques which were 1 mm thick.

### 3.4. Characterization

XRD patterns were obtained by a Pan Analytical (Philips, Almelo, The Netherland) X’Pert Pro PW1830 using CuKα radiation at a wavelength of λ = 1.54 Å. Typical power settings were 30 mA and 30 kV. The scan range was 20°–80° for composites and varistor powder with a scan rate of 2°/min. Fractured samples of the blends were coated with gold by using a Denton Vacuum sputtering unit. The FeSEM micrographs were recorded at magnifications of 10–300,000. EDX measurements were conducted by Joel-Jsm-7600F field emission SEM. Transmission electron microscopy (TEM) was performed by a Hitachi (H-7100) electron microscope with an accelerating voltage of 120 keV. The TEM polymer composite samples were prepared by dispersing approximately 0.1 g of polymer-based composite in chloroform. This dispersion was dropped on a carbon-coated copper grid and allowed to evaporate by air drying. Then, the sample was placed in the vacuum chamber for viewing. Particle size measurement and distribution graphs were done using Image Tool software and exploring the results with SPSS 20 to get the histogram plot and mean diameters of the particles. All the current-voltage measurements were taken at 0.5 V intervals up to 100 V using a source measurement unit (Kiethley, Model 236, Cleveland, OH, USA). In this study, a disk that was 10 mm in diameter and 1 mm thick was coated with silver paint on both sides for the electrical contacts in the current-voltage (I–V) measurement.

## 4. Conclusions

The electrically-resistive varistor polymer composites were fabricated successfully using the melt-blending method. The XRD pattern of the PCL, filler, and PCL/filler composite revealed the crystallinity of the composite by embedding the inorganic filler in the polymer. The TEM image showed the spherical morphology of the nano-sized filler, which improved the electrical properties of the resulting nano-composite. Meanwhile, the field emission SEM illustrated the structural properties of the nano-sized filler. An EDX analysis confirmed the formation of the phases after sintering. The I–V characteristics suggested varistor-like behavior. From the 15 wt%, 50 wt%, and 70 wt% composites, when the concentration of the nano-sized filler increased, the alpha value also increased. This could be due to the adequate amount of filler that caused the filler particles to become closer and form linkages.

## Figures and Tables

**Table 1:** Comparison of peak positions of secondary phases between nano-sized varistor powder and its polymer composite.

Ingredients	Varistor nanocomposite	Varistor nano-sized powder

	2θ (degree)	2θ (degree)
Bismuth Zinc Oxide	39.51, 45.17, 48.99, 62.91, 77.01	39.55, 63.01, 65.71, 72.73
Zinc Cobalt Oxide	49.11, 59.29, 77.08	48.99, 62.91, 74.14
Zinc Manganese Oxide	30.34, 43.69, 62.91	38.85, 56.73, 60.86, 68.08, 69.21

**Table 2:** Nonlinear coefficient for four different concentrations of polymer composites.

Composition	PCL/20 wt% filler	PCL/50 wt% filler	PCL/70 wt% filler	Pure PCL
**Alpha**	1.71	2.21	3.57	1.11
